# Supplementary material for: Local mutational diversity drives intratumoral immune heterogeneity in non-small cell lung cancer
Source: Nat Commun. 2018 Dec 18;9:5361. doi: 10.1038/s41467-018-07767-w (PMC6299138; doi:10.1038/s41467-018-07767-w)
Supplement: Supplementary file 1 — Supplementary Information [file 41467_2018_7767_MOESM1_ESM.pdf]

Supplementary Information for

Local Mutational Diversity Drives Intratumoral Immune Heterogeneity in Non-Small Cell Lung Cancer

Jia, Wu, et al.

The Supplementary file includes:

Figure S1. Workflow diagram of multi-dimensional profiling analysis.

Figure S2. Distribution of somatic mutation, predicted neoantigen and T-cell repertoire

Figure S3. Correlation between predicted neoantigen loads and T-cell repertoire or inflamed immune signature.

Figure S4. Correlation between clonal mutation burden and inflamed signatures

Figure S5. Correlation between TMB and gene panel in POPLAR trial

Figure S6. Expression of parameters used for categorization of Immunophenotype Score

Figure S7. Intra-individual heterogeneity of immunological status

Figure S8. TMB, PD-L1 mRNA expression, and PD-L1 immunohistochemical staining for P019

Figure S9. Phylogenetic architecture of non-synonymous somatic mutations

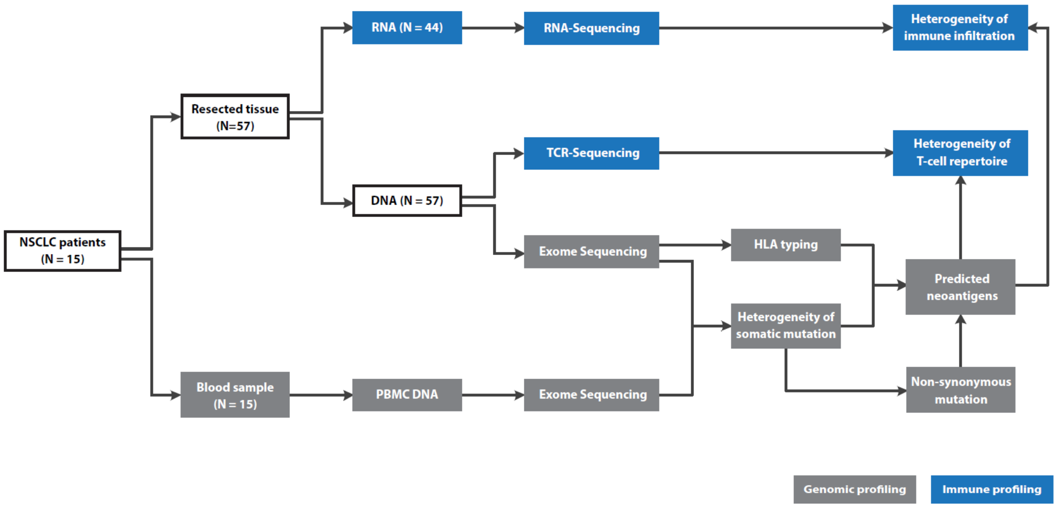

Supplementary Fig. 1 | Workflow diagram of multidimensional profiling analysis

Peripheral blood and tumor biopsies from multiple tumor loci were collected from patients (N=15). Using DNA extracted from fresh surgically-removed tumor tissues, we performed whole exome sequencing and T cell repertoire sequencing. RNA was also extracted for gene expression profiling. Genomic profiles (red) were characterized from whole exome sequencing data. Immune profiles (blue) were characterized from T cell repertoire sequencing and gene expression profiling data. Characterized genomic profiles included non-synonymous somatic mutations, HLA phenotyping, copy number alterations, and predicted neoantigens. TCR clonality was derived from the total number, frequency and supported reads of identified TCR clonotypes. Immune cell infiltration was calculated from RNA sequencing data. Bolded text indicates variables analyzed. Italics indicate software used to generate the associated variables.

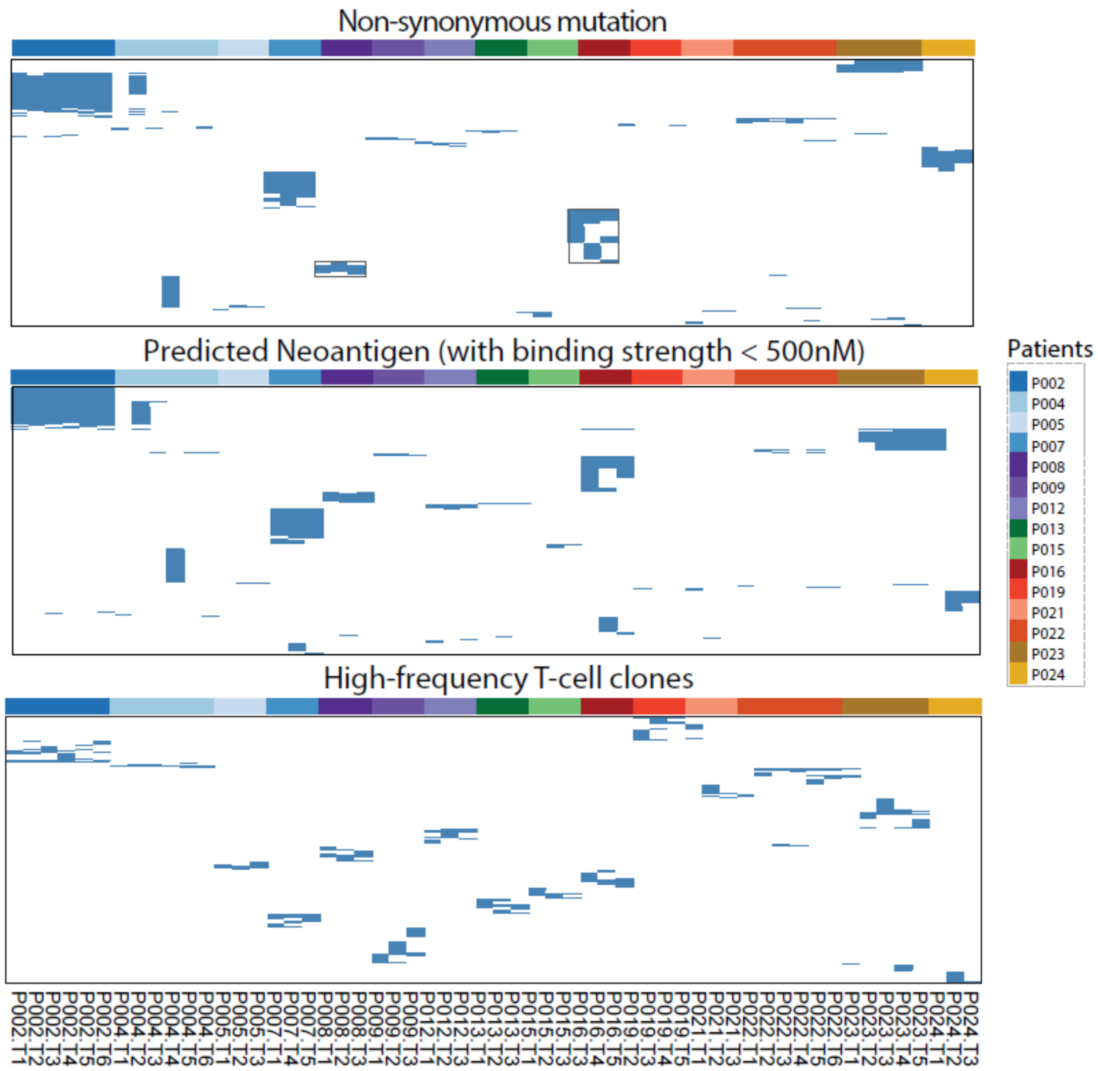

Supplementary Fig. 2 | Spatial distribution of somatic mutation, predicted neoantigen and CDR3 regions of T-cell clones for each patient

Heat maps depicting the inter-population and intra-tumoral distribution of non-synonymous mutations, predicted neoantigens (with binding strength < 500nM), and dominant T-cell CDR3 clones (frequencies ⩾ 0.5%, based on the amino acid sequence in CDR3 region) in all sequenced subjects; presence (blue) or absence (white) is indicated for every tumor focus. Samples were grouped according to individual patients and labelled in the annotation bar above. Upper panel, somatic mutation; middle panel, predicted neoantigens; lower panel, dominant T-cell CDR3 clones.

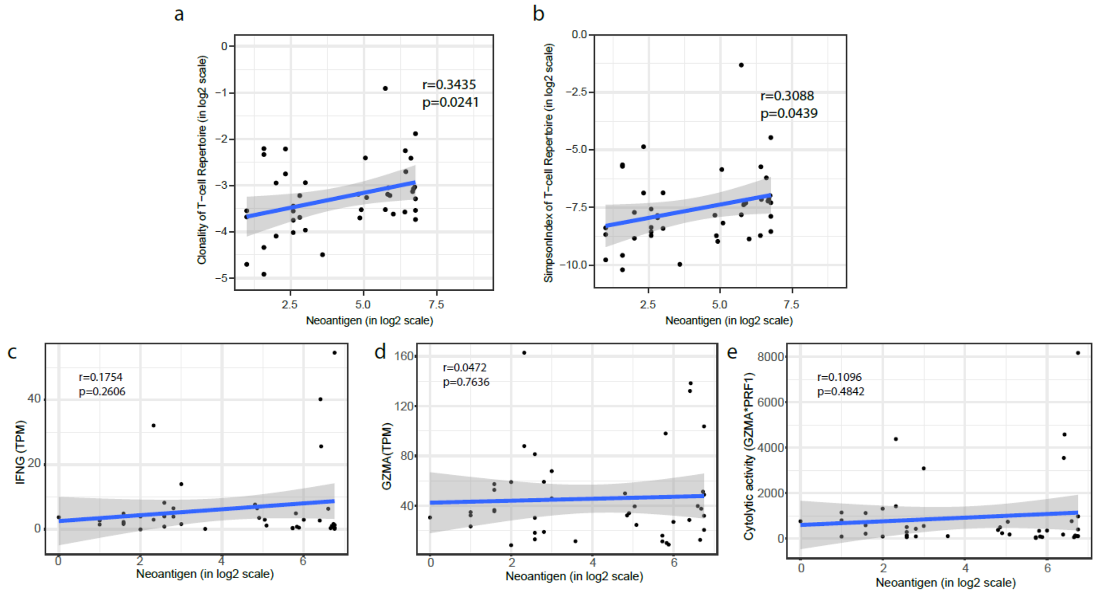

Supplementary Fig. 3 | Correlation between predicted neoantigen loads and T-cell repertoire or inflamed immune signature.

Scatterplot showing correlation between the number of predicted neoantigen (with binding strength < 50nM) and expanded properties of the T cell repertoire. Shannon entropy index of T cell clonality **(a)** and Simpson diversity index **(b)** were used to depict the T cell repertoire composition. Enrichment of highly expanded clones results in higher values for clonality and Simpson diversity. **(c-e)** Correlation between predicted neoantigen (with binding strength < 50nM) with expression of interferon-gamma, granzyme-A, and cytolytic activity (measured as the geometric mean of granzyme-A with perforin-1) in log2 of transcript per kilobase million (TPM). R, coefficient of Pearson correlation. Shaded areas represent the 95% confidence interval of fitting.

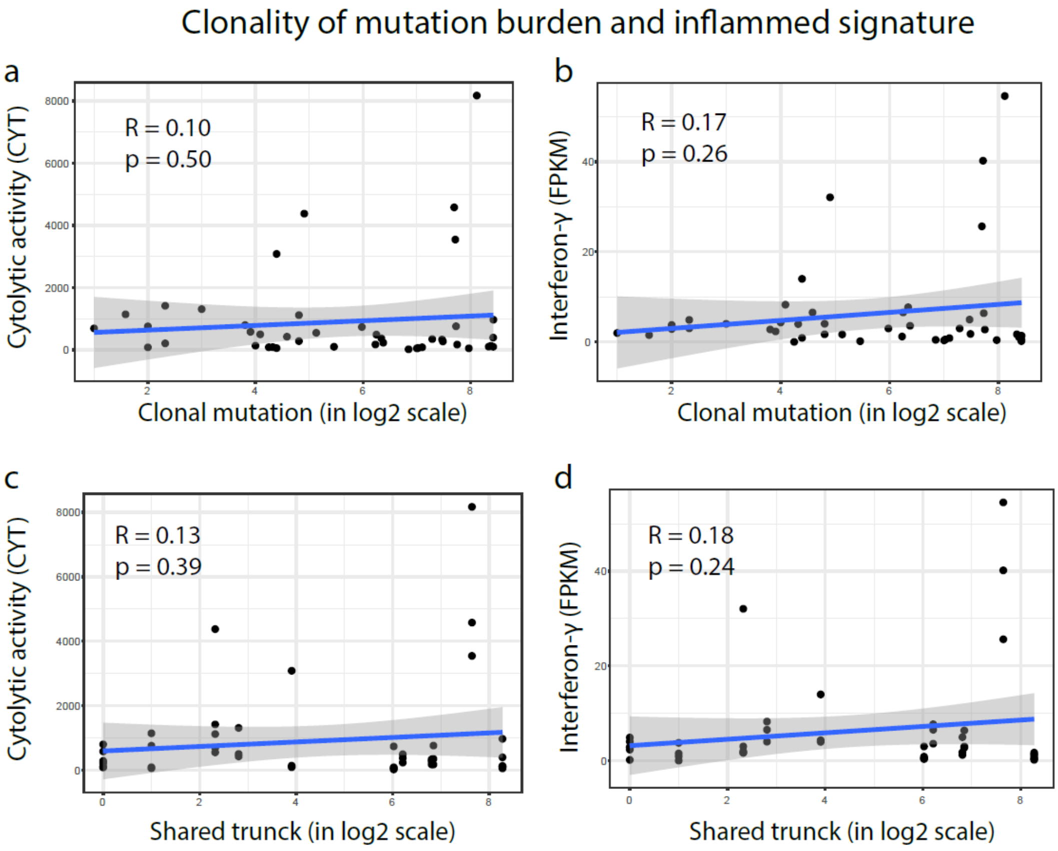

Supplementary Fig. 4 | Clonal mutation burden with T-cell inflamed signature

Correlation between clonal mutation load with expression of interferon-gamma, and cytolytic activity. Clonal mutation burden was defined as “ubiquitous and truncal” clones as previous method[1,2]. **(a, b)** Clonal mutation burden was calculated according to Blakely’s method[2]. **(c, d)** Clonal mutation burden was calculated according to McGranahan’s method[1]. CYT, the product of *PRF1* and *GZMA* expression in TPM format. R, coefficient of Pearson correlation. Shaded area represent the 95% confidence interval of fitting.

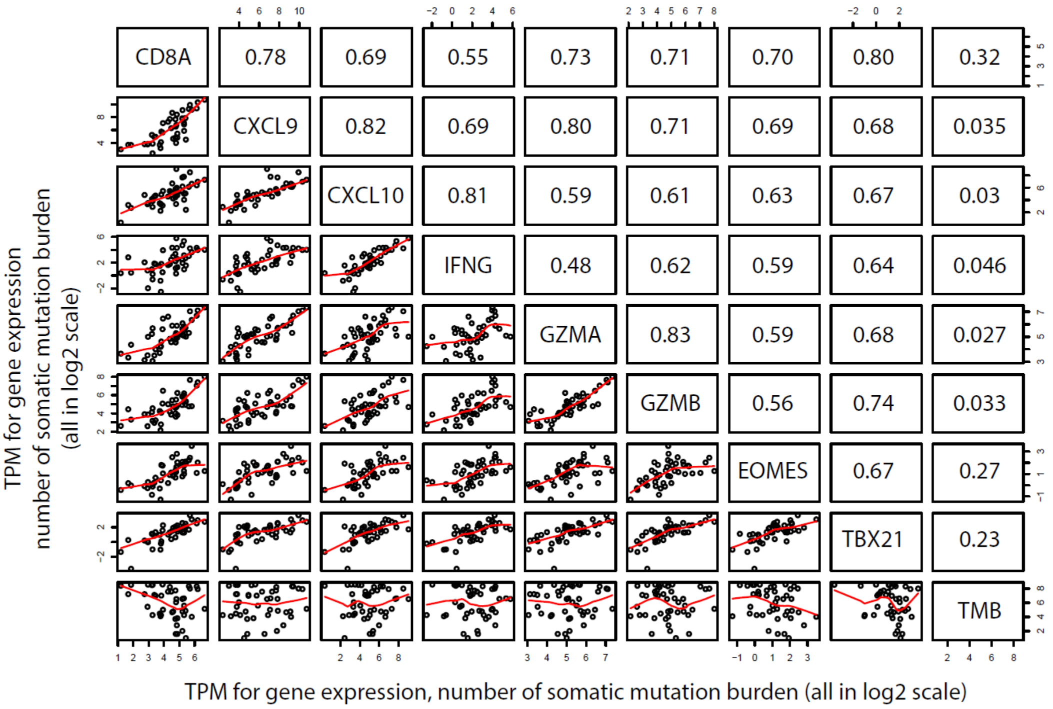

Supplementary Fig. 5 | Expression correlation of anti-PD-1-responsive gene panels with total mutation load

Correlations between the genes defining the Teff/IFNγ signature (*GZMA*, *GZMB*, *IFNG*, *EOMES*, *CXCL9*, *CXCL10*, and *TBX21*, defined by POPLAR trial previously) with mutation load and clonality, depicted as both Spearman correlation coefficient (upper triangle) and individual data points (lower triangle). Gene expression data are normalized to TPM in log2 scale. Red lines represent lines of LOESS fit.

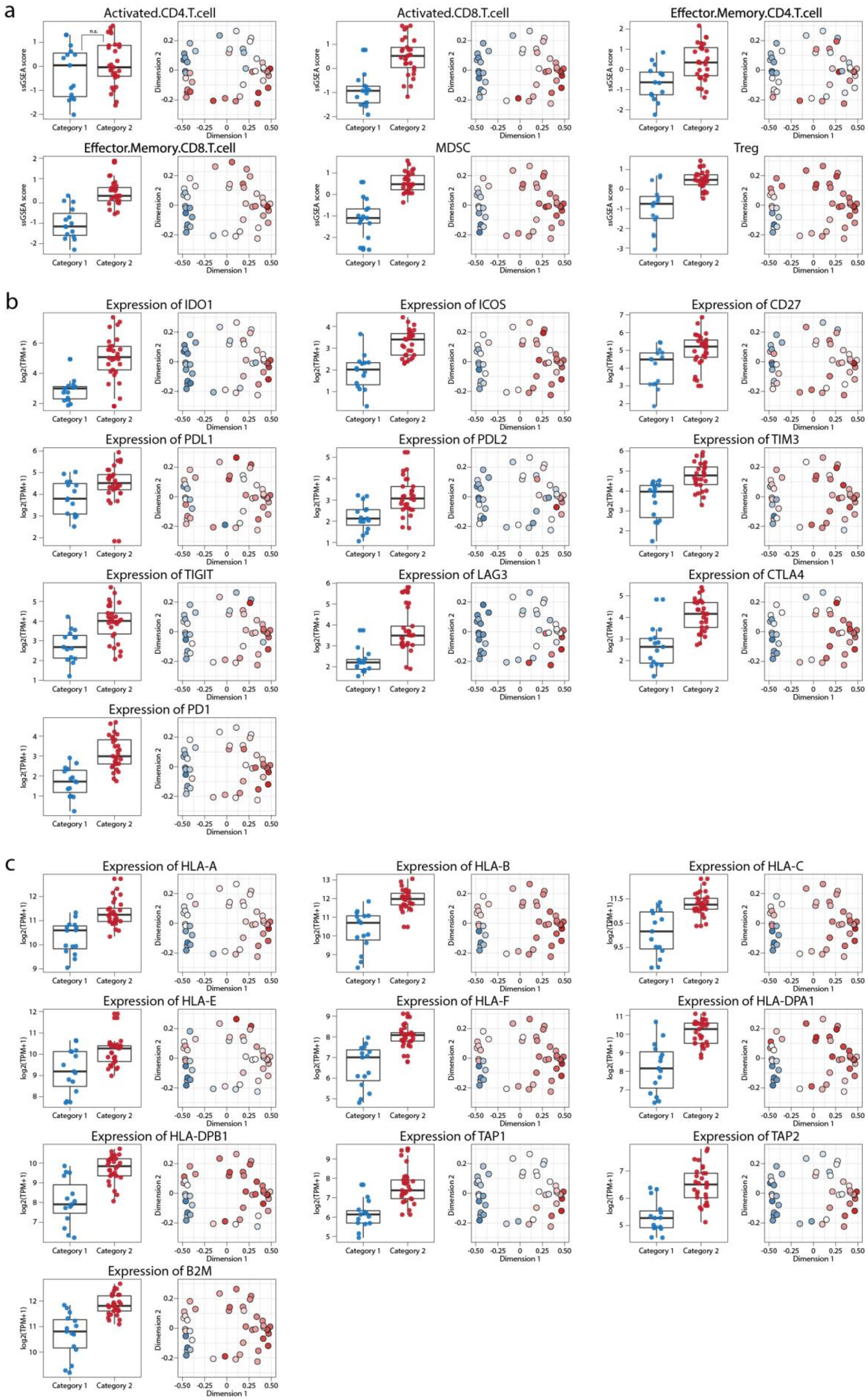
a
Activated.CD4.T.cell
n.s.
Activated.CD8.T.cell
Effector.Memory.CD4.T.cell
Effector.Memory.CD8.T.cell
MDSC
Treg
ssGSEA score
Category 1
Category 2
Dimension 1
Dimension 2
b
Expression of IDO1
Expression of ICOS
Expression of CD27
Expression of PDL1
Expression of PDL2
Expression of TIM3
Expression of TIGIT
Expression of LAG3
Expression of CTLA4
Expression of PD1
log2(TPM+1)
c
Expression of HLA-A
Expression of HLA-B
Expression of HLA-C
Expression of HLA-E
Expression of HLA-F
Expression of HLA-DPA1
Expression of HLA-DPB1
Expression of TAP1
Expression of TAP2
Expression of B2M

Supplementary Fig. 6 | Expression of parameters used for categorization of Immunophenotype Score

Visualization based on two-dimensional coordinates from multi-dimensional scaling (MDS) of proximity from the input variables used in major parameter determination for NSCLC. The color gradient indicates the parameters in immunophenotype score (IPS) for each sampling site, Red, high values; blue, lower values. Comparisons of immune cell infiltration **(a)**, immune checkpoint molecules **(b)**, and antigen presentation machinery molecules **(c)**. Statistics based on two-tailed Mann-Whitney *U*-test.

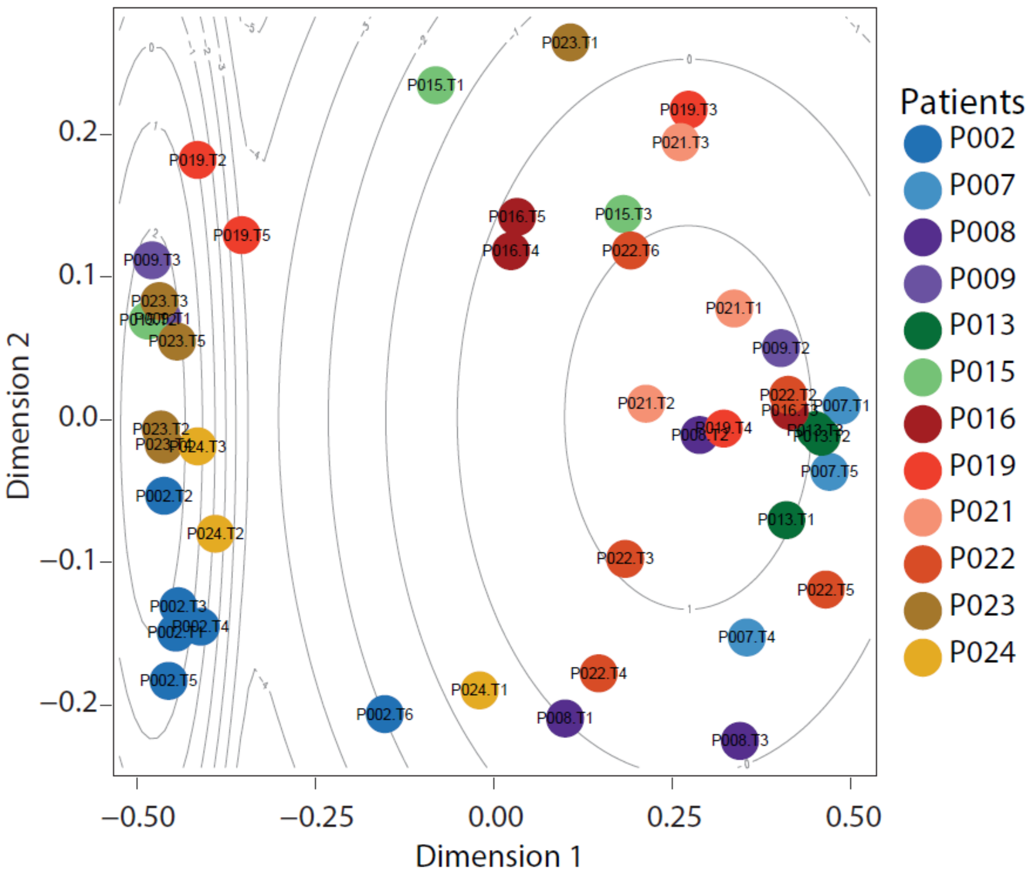

Supplementary Fig. 7 | Intra-individual heterogeneity of immunological status

Visualization based on two-dimensional coordinates from multi-dimensional scaling (MDS) of proximity matrix from the input variables in NSCLC. The Gaussian expectation maximization algorithm was employed to perform the categorization under Gaussian mixture models. The contour shows the estimated probability density for the two categories. The log-likelihood is shown. Identifiers for each sample are labelled on each dot.

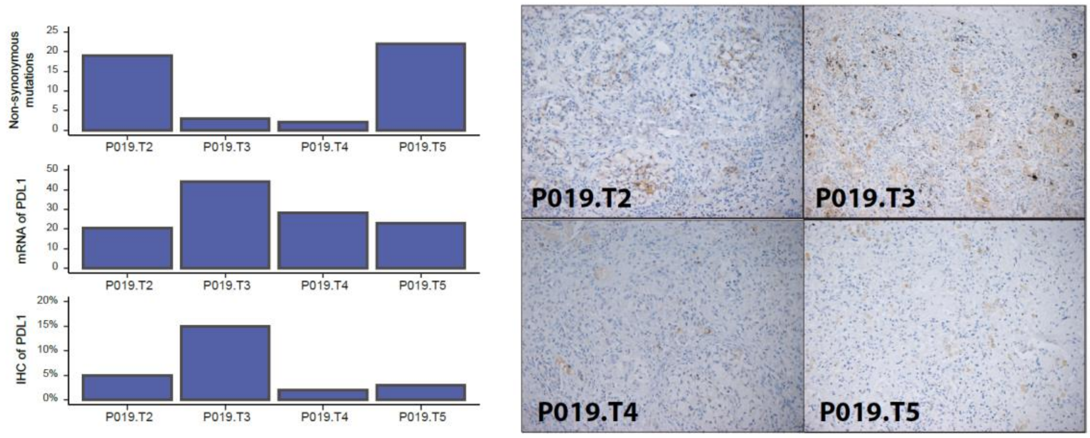

Supplementary Fig. 8 | TMB, PD-L1 mRNA expression, and PD-L1 immunohistochemical staining for P019

Correlation among TMB, PD-L1 mRNA expression, and PD-L1 protein levels are shown for Patient 019. PD-L1 immunohistochemistry was performed for each sample from P019.

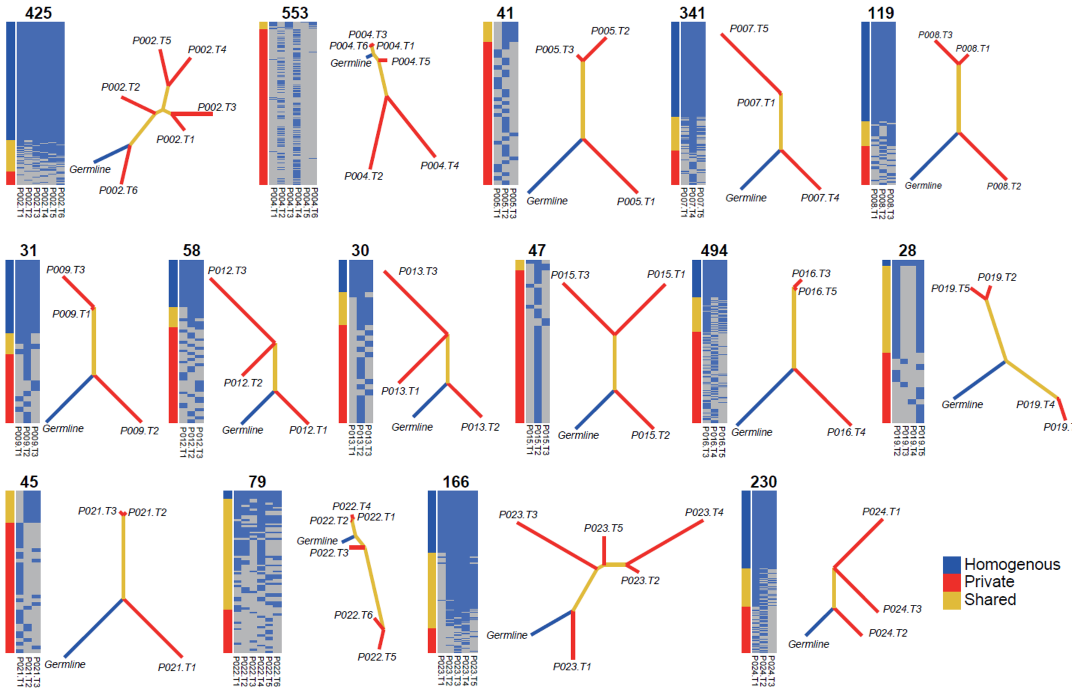

Supplementary Fig. 9 | Phylogenetic architecture of non-synonymous somatic mutations

Heatmap showing the distribution of all non-synonymous mutations; the presence (blue) or absence (gray) of each mutation is indicated for every tumor locus. The legend to the left of the heatmap shows the spatial distribution of mutations; mutation present in all tumor loci (homogenous, blue), shared in more than one but not all loci (shared, yellow), and in only one tumor locus (private, red). The total number of non-synonymous mutations is provided for each tumor on the above of heatmap. Phylogenetic trees of all detected mutations are shown to the right of the heatmap; lengths of trunks and branches represent the number of non-synonymous mutations acquired.

References

1. McGranahan, N., *et al.* Clonal neoantigens elicit T cell immunoreactivity and sensitivity to immune checkpoint blockade. *Science.* **351**, 1463-1469. doi: 1410.1126/science.aaf1490. Epub 2016 Mar 1463. (2016).
2. Blakely, C.M., *et al.* Evolution and clinical impact of co-occurring genetic alterations in advanced-stage EGFR-mutant lung cancers. *Nat Genet.* **49**, 1693-1704. doi: 1610.1038/ng.3990. Epub 2017 Nov 1696. (2017).
