# Supplementary material for: Local mutational diversity drives intratumoral immune heterogeneity in non-small cell lung cancer
Source: Nat Commun. 2018 Dec 18;9:5361. doi: 10.1038/s41467-018-07767-w (PMC6299138; doi:10.1038/s41467-018-07767-w)
Supplement: Supplementary file 13 — Description of Additional Supplementary Files [file 41467_2018_7767_MOESM13_ESM.docx]

**Title:** Supplementary Table S1
**Description:** Characteristics of Patients

**Title:** Supplementary Table S2
**Description:** Cellular purity of tumor cells

**Title:** Supplementary Table S3
**Description:** WES Summary

**Title:** Supplementary Table S4
**Description:** WES Detailed

**Title:** Supplementary Table S5
**Description:** TCR dominant

**Title:** Supplementary Table S6
**Description:** TCR detected

**Title:** Supplementary Table S7
**Description:** TCR Clonality

**Title:** Supplementary Table S8
**Description:** Predicted neoantigen

**Title:** Supplementary Table S9
**Description:** Input variables for randomForest

**Title:** Supplementary Table S10
**Description:** TPM

**Title:** Supplementary Table S11
**Description:** Immune cell infiltration Supplementary Table S12 - BIOCARTA ssGSEA score
